# Characterization of Macrophage and Cytokine Interactions with Biomaterials Used in Negative-Pressure Wound Therapy

**DOI:** 10.3390/bioengineering9010002

**Published:** 2021-12-22

**Authors:** Praveen Krishna Veerasubramanian, Victor C. Joe, Wendy F. Liu, Timothy L. Downing

**Affiliations:** 1 Department of Biomedical Engineering, University of California-Irvine, Irvine, CA 92697, USA; pveerasu@uci.edu; 2 UCI Edwards Lifesciences Foundation Cardiovascular Innovation and Research Center (CIRC), University of California-Irvine, Irvine, CA 92697, USA; 3 Department of Surgery, University of California-Irvine, Irvine, CA 92697, USA; vcjoe@hs.uci.edu; 4 Department of Chemical and Biomolecular Engineering, University of California-Irvine, Irvine, CA 92697, USA; 5 Institute for Immunology, University of California-Irvine, Irvine, CA 92697, USA; 6 Department of Molecular Biology and Biochemistry, University of California-Irvine, Irvine, CA 92697, USA; 7 NSF-Simons Center for Multiscale Cell Fate Research, University of California-Irvine, Irvine, CA 92697, USA; 8 Department of Microbiology and Molecular Genetics, University of California-Irvine, Irvine, CA 92697, USA

**Keywords:** macrophages, negative-pressure wound therapy, wound healing, wound dressings, cell–material interactions

## Abstract

Macrophages are innate immune cells that help wounds heal. Here, we study the potential immunomodulatory effects of negative-pressure wound therapy (NPWT) materials on the macrophage inflammatory response. We compared the effects of two materials, Granufoam™ (GF) and Veraflo Cleanse™ (VC), on macrophage function in vitro. We find that both materials cause reduced expression of inflammatory genes, such as TNF and IL1B, in human macrophages stimulated with bacterial lipopolysaccharide (LPS) and interferon-gamma (IFNγ). Relative to adherent glass control surfaces, VC discourages macrophage adhesion and spreading, and may potentially sequester LPS/IFNγ and cytokines that the cells produce. GF, on the other hand, was less suppressive of inflammation, supported macrophage adhesion and spreading better than VC, and sequestered lesser quantities of LPS/IFNγ in comparison to VC. The control dressing material cotton gauze (CT) was also immunosuppressive, capable of TNF-α retention and LPS/IFNγ sequestration. Our findings suggest that NPWT material interactions with cells, as well as soluble factors including cytokines and LPS, can modulate the immune response, independent of vacuum application. We have also established methodological strategies for studying NPWT materials and reveal the potential utility of cell-based in vitro studies for elucidating biological effects of NPWT materials.

## 1. Introduction

Negative-pressure wound therapy (NPWT) is a clinically used treatment that is indicated for a variety of conditions including chronic wounds and burns, utilizing form-fitting sponge dressings and vacuum application to aid in healing. The treatment applies suction on the wound bed that has been packed with dressing and sealed airtight, producing a local hypoxia and removing wound exudates. Mechanical strain caused by NPWT application is thought to stimulate cellular proliferation while local hypoxia enhances angiogenesis [1]. NPWT can also ameliorate inflammation by providing edema relief, exudate removal, and bioburden reduction, all leading to a reduction in proinflammatory factors and proteases build-up [2]. However, NPWT treatment still fails in a significant percentage (29%) of patients, associated with morbidities, such as atherosclerosis of the lower extremities, diabetes, peripheral vascular disease wounds, pressure ulcers, and infections of *Staphylococcus aureus* and *Pseudomonas aeruginosa* [3]. Beyond gross tissue analysis, relatively little has been explored regarding the molecular and cellular mechanisms involved during NPWT. Mechanistic studies that precisely characterize the impact of NPWT on critical biological processes during wound healing are warranted to develop therapeutic strategies that can improve patient outcomes. Specifically, understanding how commercial biomaterials used in NPWT modulate phenotypes of immune cells, such as macrophages, in dermal wounds is crucial.

Macrophages are mechanosensitive innate immune cells that are key regulators of wound healing and foreign body responses [4,5]. While discharging their various functions in the body, which range from homeostasis to inflammation and tissue regeneration, these cells encounter diverse microenvironmental cues that are thought to impact their function by altered cellular shape, spread, focal adhesion, and force generation [6,7,8,9]. Macrophages are among the early responders to injury sites, appearing between 24 and 48 h after injury [10], and often encountering a compliant microenvironment in the form of a fibrin clot [11]. External wounds are accompanied by the presence of invading pathogens and neutrophil signaling, which provoke a proinflammatory phenotype in macrophages, unleashing free radicals and phagocytosis-mediated immunity. As the wound heals and the ECM secreted by proliferating fibroblasts mature, the collagen-rich ECM stiffens. Several studies have shown that compliant substrates can evoke a subdued inflammatory response from macrophages in comparison to stiffer substrates [12,13]. Stiffer substrates elicit increased macrophage spread, actin maturity, and the degree of activation in response to bacterial lipopolysaccharides (LPS) [13]. Other cues, such as ECM architecture, mechanical stretch, shear and compressive forces, or fluid flow, have also been described to alter macrophage response to biochemical stimuli [8]. External wounds are not just subject to an evolving microenvironment, but also witness the application of medical aids, such as wound dressings, that interact intimately with the wound sites. The recognition of the substantial macrophage contributions to wound healing has led to wound dressings whose development was motivated solely by macrophage immunomodulation [14].

Despite the prominent roles of macrophages in inflammation and wound healing, macrophage immunomodulation during NPWT remains largely undescribed. NPWT has been demonstrated to lower proinflammatory TNF-α and IL-1β clinically [15], and enhance pro-healing TGF-β and IL-10 [16], cytokines that are usually attributed to macrophages. This can be explained by the significantly reduced CD68+ macrophages observed during NPWT therapy [15]. A recent study explored the roles of macrophages in NPWT-mediated healing, reporting lower macrophage autophagy in NPWT-treated diabetic wounds [17]. Additionally, application of vacuum on Raw264.7 macrophages in vitro resulted in lower inflammatory activation and autophagy [17], which is expected considering the immunomodulatory influences that hypoxia has long been known to exert on macrophages within tumor niches [18]. These data point towards NPWT modulating chronic inflammatory activation in macrophages at wound sites to advance healing. However, there is little understanding of how the NPWT materials themselves impact macrophage inflammatory activation.

The dressings used in NPWT are microporous synthetic polymers of varied porosity and hydrophobicity, whose mere application (without suction) could alter cell phenotype and function, impacting the clinical outcome. For instance, one study that utilized a diabetic mouse wound model found that the density of blood vessels was higher in case of NPWT foam application, in comparison to occlusive dressing controls and vacuum application without foam placement [19]. Taking note of the mechanoresponsive nature of macrophages, we asked if the NPWT materials utilized in the clinic could be immunomodulatory, independent of vacuum application. In this study, we used two different NPWT foams that display a pore volume fraction of between 0.8 and 0.9. The first is V.A.C.^®^ Granufoam™, which is a hydrophobic polyurethane ether open cell reticulated foam, with a pore size ranging from 400–600 μm. It is utilized for general purpose vacuum-assisted closure, where the dressing may be changed every 24–72 h. The second is V.A.C.^®^ Veraflo Cleanse™, which is a relatively hydrophilic polyurethane ester open cell reticulated foam, with a pore size ranging from 133–600 μm. It is utilized for NPWT with instillation, where the wound bed may be irrigated with saline supplemented with topical medications. The irrigant may be removed by suction after a defined dwell time (typically 10 min [20]). NPWT with instillation is aimed at removing bioburden more effectively than regular NPWT [21]. Both these materials may be used without a secondary wound contact layer as bridging therapy to induce healing by contraction in large and acute wounds (for instance, sternotomy [22] and fasciotomy in limb compartment syndromes [23]). Given that previous studies (including ours) have shown that macrophage adhesion and interaction with biomaterials can modulate their inflammatory response [12,13,24,25,26], we hypothesized that these NPWT materials, chosen for their diverse purposes and properties, could evoke differential activation of macrophages. We examined macrophage responses when cultured in the presence of these two materials, in comparison to cotton gauze (wound dressing/contact layer [27]), glass (cell adhesive surface), and ultralow attachment surfaces (non-adhesive surface) as controls. To stimulate macrophages towards a proinflammatory phenotype (commonly referred to as “M1”), we added LPS and IFNγ, and then examined the expression of inflammatory genes, such as *TNF*, and *IL1B*, and the chemoattractant *CCL2*. Upon finding both NPWT materials to be suppressive of inflammatory activation in THP-1 human macrophages (albeit to varying degrees) during direct cell-material culture, we also studied the potential of these materials to sequester biochemical molecules that enhance the macrophage proinflammatory phenotype through paracrine or autocrine signaling.

## 2. Materials and Methods

### 2.1. Cell Culture and Media

THP-1 cells purchased from ATCC (Cat. TIB-202) were cultured in RPMI-1640 medium (Gibco, Cat. A1049101), supplemented with 10% FBS, 1x Pen/Strep, and 50 μM β-mercaptoethanol. Then, 20 nM of phorbol 12-Myristate 13-Acetate (PMA) (Sigma, Cat. 19-144) was used to induce differentiation of THP-1 monocytes to macrophages in 48 h. The NPWT materials were cut to 15 mm discs of approximate 1 mm thickness, and cells were seeded on them in 24-well ultralow attachment plates at a density of 250,000 cells in 2 mL of media per well. The NPWT materials used for this study were Granufoam™ (GF, a polyurethane ether) and Veraflo Cleanse™ (VC, a polyurethane ester) (both from 3M™ V.A.C.^®^), while cotton (CT) (Fisherbrand Cat. 22-415-468) was used as a traditional dressing and wound contact layer control. Ultralow attachment plates (UL) (Corning Costar Cat. CLS3473) were used for the cell culture, to discourage cells from adhering to and spreading on the bottom of the dishes. Further, 15 mm glass (GL) coverslips were used as control surfaces for adherent cell culture wherever indicated. Direct cell-material culture experiments had THP-1 cells added onto the materials with PMA addition. At 48 h of PMA-mediated differentiation, THP-1 macrophages were stimulated towards a proinflammatory phenotype by the addition of 10 ng/mL LPS (Invivogen Cat. tlrl-3pelps) and IFNγ (Peprotech, Cat. 300-02), without removal of the existing media or non-adherent cells. After stimulation for a period of 6 h before the media supernatant was collected for ELISA, the cells in the material were lysed in Trizol following media aspiration using a pipette.

### 2.2. Transwell Culture

Transwell experiments were performed by culturing cells at the bottom of tissue culture dishes, with the NPWT materials placed on transwell inserts comprised of a PET membrane with 8 μm pores (Corning Falcon Cat. 353097).

### 2.3. Cell Viability Assay

Cell viability was measured using an MTT (3- (4,5-dimethylthiazol-2-yl)-2,5-diphenyltetrazolium bromide) assay [28]. Briefly, cells were seeded by scaling down the cell-material culture experiments to a 96-well format (Corning Costar Cat. CLS3474). Regular tissue culture plates were also used as a control. THP-1 cells were cultured with PMA for 48 h on the NPWT materials. Following this, MTT reagent was added to a final concentration of 0.5 mg/mL. After 4 h of incubation at 37 °C, 100 µL of solubilization solution (16% SDS in 40% dimethylformamide and 2% acetic acid) were added and mixed well for 30 min. Post solubilization, the NPWT materials were squeezed and removed, and the plate was read using a microplate reader at 570 nm. % viability was measured as ((Abs_sample_ − Abs_blank_)/(average Abs_control_ − Abs_blank_)) × 100.

### 2.4. LPS/IFNγ and TNF-α Cytokine Sequestration Studies

The LPS/IFNγ sequestration capabilities of the materials were measured by preincubating the materials with THP-1 media containing LPS/IFNγ for 1 h and then transferring the media to THP-1 macrophages differentiated with PMA for 48 h on regular tissue culture plates. The media supernatant was collected after 6 h to study the response of cells to the transferred media and understand the LPS/IFNγ sequestering potential of each material.

The potential of these materials to sequester TNF-α cytokine produced by the macrophages in response to proinflammatory stimulation was studied by transferring the inflammatory cytokine-containing media produced by macrophages in response to LPS/IFNγ, onto NPWT materials for a period of 1 h, followed by an ELISA of the media supernatant.

### 2.5. ELISA on Media Supernatant and on NPWT Materials

TNF and MCP-1 ELISA were performed using kits following the manufacturer’s recommendations (Biolegend Cat. 430201, 438804). ELISA assays on the biomaterials were performed by scaling down the cell-material culture experiments to a 96-well format (Corning Costar Cat. CLS3474). The media was removed from the materials by aspirating out the media and blocking the materials with 1% bovine serum albumin. Following this, ELISA was performed on the materials as a substrate by adding detection antibody, Avidin-HRP, and TMB substrate sequentially from the same kit as above.

### 2.6. RNA Extraction and qRT-PCR

Cells were lysed with Trizol reagent (Sigma), and their RNA extracted using a Direct-zol RNA extraction kit (Zymo Research #R2051), and reverse transcribed to cDNA using a High-Capacity cDNA Transcription Kit (Thermo Fischer Scientific #4368814). This was then used for quantitative PCR (qPCR). Samples were diluted and analyzed using PerfeCTa SYBR Green SuperMix (Quantabio Cat. 95054-500). Primer concentrations were kept at 300 nM and 2 step qPCR was performed as described according to the manufacturer’s protocol. The primers used for qRT-PCR are as follows: TNF—AGGCGCTCCCCAAGAAGACAGG (Forward), CAGCAGGCAGAAGAGCGTGGTG (Reverse); CCL2—CCCCAGTCACCTGCTGTTAT (Forward), TGGAATCCTGAACCCACTTC (Reverse); IL1B—CCGACCACCACTACAGCAAG (Forward), GGGCAGGGAACCAGCATCTT (Reverse); and RPL37A—ATTGAAATCAGCCAGCACGC (Forward), AGGAACCACAGTGCCAGATCC (Reverse). RPL37A was used as a housekeeping gene for normalization.

### 2.7. Determination of Cell Distribution in Culture on the Materials

The cell populations on the materials were measured by estimating the cellular content in the media supernatant and on the ultralow attachment plate surfaces using a nucleic acid quantification assay (CyQUANT NF, Thermo, Cat. C35007) as per the manufacturer’s instructions. Briefly, lysis buffer with the CyQUANT reagent were added to the cells/cell pellet and incubated for 30 min. This was read using a fluorescent plate reader (Thermo Fluoroskan Ascent FL) at an excitation of 485 nm and emission of 527 nm. A standard curve was created using known numbers of THP-1 cells to establish the relationship between the readout and the cell population.

### 2.8. Calorimetric Staining

Cell adhesion to the NPWT materials was confirmed by staining for a human nuclear marker. Briefly, the materials were seeded with THP-1 cells and fixed with 4% paraformaldehyde, permeabilized with 0.3% Triton-X100 for 10 min, blocked with 2% bovine serum albumin, and stained with Rabbit Ku80 antibody (1:500 dilution, Cell Signaling, Cat. 2180S) overnight at 4 °C. After 3 washes with 2% BSA, the samples were stained with Goat Anti-Rabbit IgG H&L HRP polymer (Abcam, Cat. ab214880) for 1 h at room temperature. Finally, the samples were developed using a DAB substrate kit (Abcam, Cat. ab64238) for 5 min, washed, and mounted using Fluoromount-G (Southern Biotech) for visualization using a confocal microscope (Olympus Fluoview FV3000).

### 2.9. Scanning Electron Microscopy

THP-1 cells seeded on the NPWT materials were fixed with 1% paraformaldehyde for 10 min. After 3 washes with PBS, the samples were dehydrated with an ethanol gradient of increasing concentration (50%, 70%, 95%, 2 × 100%, 10 min each), and air dried. The samples were then mounted using carbon tape, and sputter coated with 5 nm of Ir. Subsequently, the samples were imaged at 30 kV using an SEM (FEI Quanta 3D-FEG).

### 2.10. Statistical Analyses

One-way ANOVA with multiple comparisons was performed on GraphPad Prism (v9, GraphPad software). All data are represented as mean ± S.E.M. in the bar plots. * indicates *p* < 0.05.

## 3. Results and Discussion

### 3.1. NPWT Materials Elicit Different Macrophage Adhesion and Attenuate Inflammatory Gene Activation

**Figure 1:**
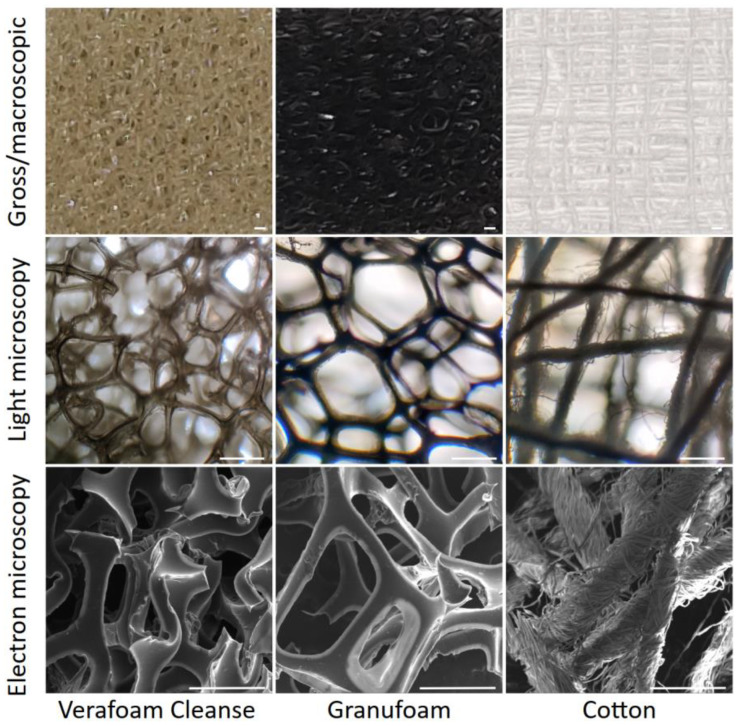
Architecture of the NPWT materials. Macroscopic (**top**), microscopic (**middle**), and electron microscopic (**bottom**) views of the three different materials studied. Scale bars are 500 μm.

In our study, we sought to examine the impact of two NPWT materials (properties summarized in Figure S1) on macrophage adhesion and inflammatory activation (relative to each other and traditional cotton wound dressing). Scanning electron microscopy (SEM) revealed that NPWT materials were highly macroporous open-reticulated foams (with 80–90% porosity). These materials provide cells with a large surface area to attach to in three dimensions, in comparison to planar culture dish surfaces. CT also has a large specific surface area [29], explained by its intricately woven fibers (Figure 1). To understand the differences in the ability of macrophages to adhere to these materials, we seeded THP-1 cells onto two different NPWT materials, Granufoam™ (henceforth referred to as GF) and Veraflo Cleanse™ (henceforth VC), as well as on traditional cotton gauze (CT) dressing. Glass coverslips (GL) were used as a cell-adhesive surface supporting robust macrophage attachment, spreading, and inflammatory activation [12]. All cell-material cultures were performed in ultralow attachment (UL) wells to minimize cell binding to the well surface.

**Figure 2:**
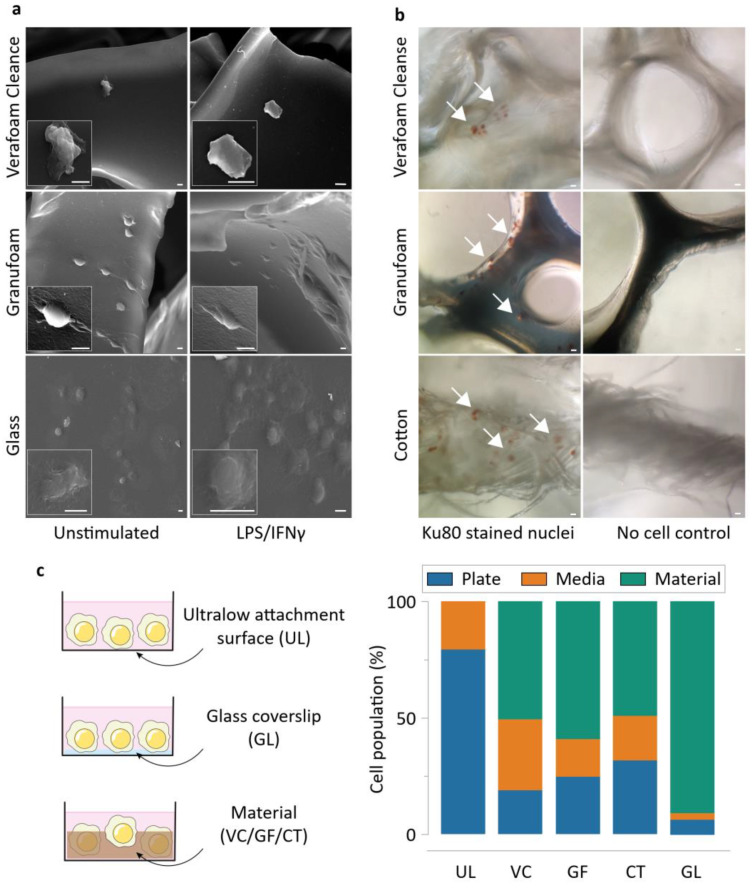
Macrophages display distinct morphology and attachment on the NPWT materials during direct culture. (**a**) Electron microscopy of representative cells attached onto the different materials. Scale bars are 5 μm. (**b**) Representative images of Ku80-stained THP-1 macrophages on the different materials, along with respective no cell controls. (**c**) Schematics of the direct culture modalities on the different surfaces (left), and CyQUANT assay results displaying the proportion of THP-1 macrophages attaching onto the materials in the study during direct culture (mean of 3 independent experiments). Abbreviations are as follows: UL—Ultralow attachment plate surface, VC—Veraflo Cleanse, GF—Granufoam, CT—Cotton gauze, GL—Glass coverslip.

Two days following cell seeding, we performed electron microscopy on the materials cultured with THP-1 cells. As expected, macrophages attached and spread well to 2D glass surfaces. While GF-supported macrophages attached and spread well, VC featured macrophages with atypical morphology (Figure 2a). VC also had a sparse cell abundance, possibly explained by an inability of the cells to adhere and spread well on it. To confirm that the cells were indeed directly attached to the materials, we stained macrophages for a human nuclear marker Ku80. We observed significant cell presence in all three materials, confirming a direct physical interaction between the cells and the materials (Figure 2b). The differences in cell adhesivity may also be explained by the material hydrophobicity, mediated by protein absorption on the material surfaces [30,31,32]. GF is comparatively hydrophobic while VC is relatively hydrophilic [33]. CT is also hydrophilic, which explains its large water retention capability [34]. To study the possibility of varying levels of cell attachment between the different groups, we quantified the cellular population using CyQUANT assays. The assay was used to measure the DNA content in the media and plate surfaces after removing the material scaffolds (and the cells attached to them). This allowed us to identify the relative proportion of cells within each culture condition that were either suspended in the media or tethered to the ultralow attachment plate surfaces, and by subtracting from the total cell population, quantify the cells attached to the material (Figure S2). We found that GF supported more cells on it than VC does, reinforcing our observations that cells do not adhere well on VC (Figure 2c). In addition, these materials were well tolerated by the macrophages, as indicated by greater than 80% viability across conditions during cytotoxicity measurements through MTT assays (Figure S1).

**Figure 3:**
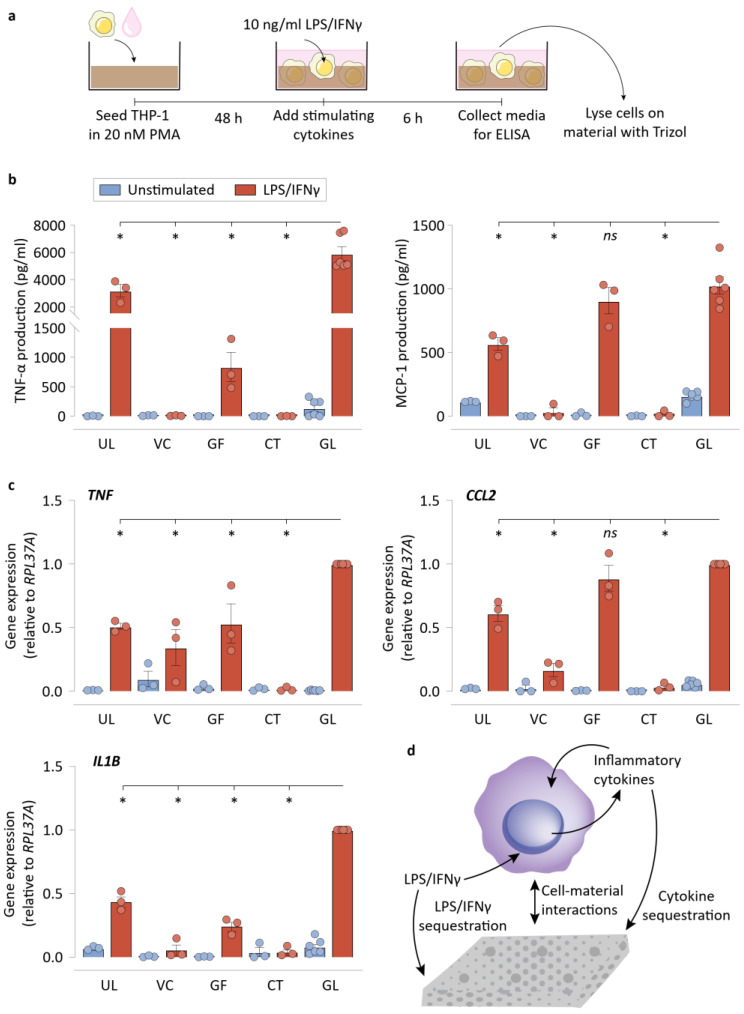
Macrophages display lower inflammatory polarization on the NPWT materials during direct culture. (**a**) Schematic of the direct culture experiments, explaining the direct culture setup and the workflow. (**b**) ELISA results of the direct culture experiments for TNF-α, and MCP-1 production by the macrophages. (**c**) qRT-PCR results of the direct culture experiments for *TNF*, *CCL2*, and *IL1B* by the macrophages. *RPL37A* was used a housekeeping gene for normalization. (**d**) Schematic depicting the various possible biochemical and biomechanical interactions between the material surfaces and the cells. All bar plots are represented as the mean of 3 or 6 independent experiments, with error indicating the S.E.M. Abbreviations are as follows: UL—Ultralow attachment plate surface, VC—Veraflo Cleanse, GF—Granufoam, CT—Cotton gauze, GL—Glass coverslip. * indicates *p* < 0.05 as tested using one-way ANOVA with multiple comparisons.

We next examined the inflammatory potential of macrophages grown in direct contact with NPWT materials (Figure 3a). Using our cell-material culture set-up described above, we found that there was significantly reduced TNF-α detectable from the cells that were seeded on UL, VC, GF, and CT in comparison to GL, when stimulated with LPS/IFNγ (Figure 3b). We also found that detected MCP-1 was significantly lower on UL, VC, and CT in comparison to GL. Gene expression of TNF and CCL2 matched the findings of the protein-level ELISA experiments, where LPS/IFNγ-stimulated macrophages on UL, VC, GF, and CT expressed less *TNF*, while macrophages on UL, VC, and CT experienced reduced *CCL2* expression compared to GL (Figure 3c). In addition, the expression of *IL1B* was also suppressed in all conditions in comparison to GL controls. Taken together, these data suggest that culturing macrophages on these materials resulted in reduced inflammatory polarization. Additionally, the *TNF* and *IL1B* downregulation experienced by macrophages seeded on UL surfaces in comparison to attachment permissive GL surfaces emphasizes the importance of macrophage attachment and spreading on a substrate to its polarization state. We posited that there are at least three possible interactions that may explain the reduced expression of inflammatory genes observed in macrophages cultured in the presence of NPWT materials: (i) physical cell-material exchanges that modulate the macrophage phenotype [8]; (ii) LPS/IFNγ sequestration by the material by adsorption (and possibly absorption by CT), leading to reduced availability of free LPS to interact with TLR4 (or IFNγ with IFN) receptors; and (iii) sequestration of cytokines that the cells produce that facilitate amplification of the inflammatory response by acting as supplemental autocrine/paracrine stimuli [35] (Figure 3d). Thus, we next set out to distinguish the impact of the said contributions of NPWT materials on macrophage behavior in the forthcoming experiments.

### 3.2. NPWT Materials Do Not Require Direct Contact with Cells to Suppress Inflammatory Activation

**Figure 4:**
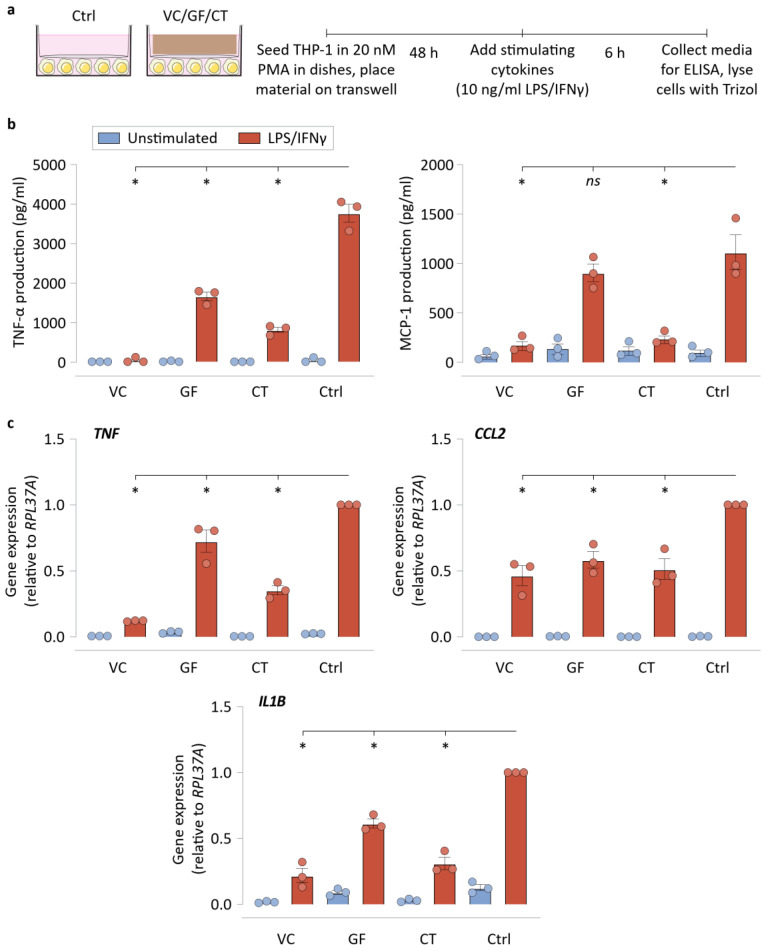
NPWT materials do not require direct contact with cells to suppress inflammatory activation. (**a**) Schematic of the transwell experiments, explaining the indirect culture setup and the workflow. (**b**) ELISA results of the transwell culture experiments for TNF-α and MCP-1 production by the macrophages. (**c**) qRT-PCR results of the transwell culture experiments for *TNF*, *CCL2*, and *IL1B* by the macrophages. *RPL37A* was used a housekeeping gene for normalization. Both experiments used empty transwell (no material) controls. All bar plots are represented as the mean of 3 independent experiments, with error indicating the S.E.M. Abbreviations are as follows: VC—Veraflo Cleanse, GF—Granufoam, CT—Cotton gauze, Ctrl—No material control. * indicates *p* < 0.05 as tested using one-way ANOVA with multiple comparisons.

We next focused on understanding the effects of mere NPWT material presence in the cell culture on macrophage polarization. We avoided direct interactions between macrophages and the NPWT materials by using a transwell setup that seeded cells in the bottom compartment, and placed NPWT materials on the top compartment (Figure 4a). We found that this indirect culture setup caused reduced TNF-α production in VC, GF, and CT conditions, in comparison to the no material control (Ctrl), implying that the material may modulate macrophage polarization even without direct contact with the materials (Figure 4b). Similarly, MCP-1 production was also dampened by the presence of VC and CT in the culture transwell inserts. We then analyzed the gene expression of *TNF*, *CCL2*, and *IL1B*, in the same transwell setup, finding that lower gene expression occurred in the presence of all three materials in comparison to Ctrl. These results show that the NPWT materials can suppress inflammatory output from LPS/IFNγ-stimulated macrophages without requiring direct physical contact. We hypothesized that this could be made possible by LPS/IFNγ sequestration or cytokine sequestration by the materials, and the next set of experiments explored the capabilities of the materials to do so. These results, however, do not undercut the significance of cell–material interactions in shaping macrophage phenotype, as was reflected in our comparisons between UL and GL surfaces in the previous figure (Figure 3b,c).

### 3.3. Sequestration of Biochemical Signals by NPWT Materials Results in Reduced Macrophage Inflammatory Activation

**Figure 5:**
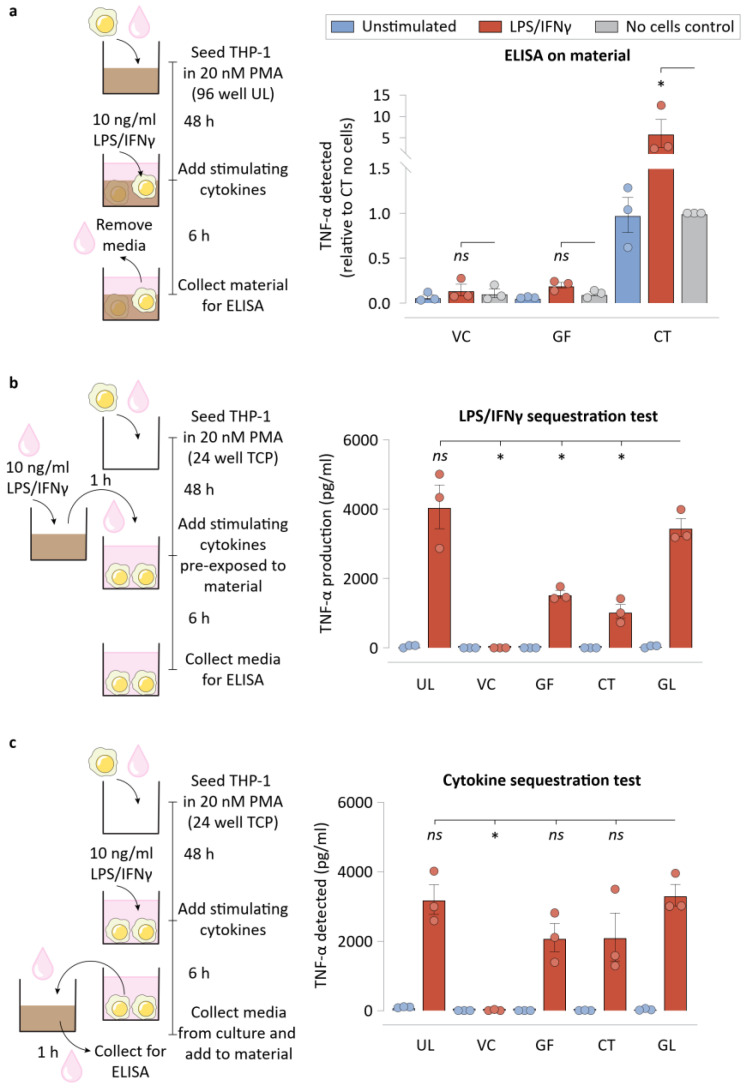
Biochemical signal sequestration by NPWT materials drives lower macrophage inflammation. (**a**) Schematic of the experimental workflow and TNF-α detected on the NPWT materials when ELISA was performed on the materials subsequent to direct culture of macrophages on them. No cell controls were included for each material condition. (**b**) Schematic of the experimental workflow for the LPS/IFNγ sequestration assay, and ELISA data showing TNF-α production by cells exposed to LPS/IFNγ that was pre-incubated on the NPWT materials for 1 h before macrophage stimulation. (**c**) Schematic of the experimental workflow for the cytokine sequestration assay, and ELISA data showing TNF-α detected in the media after 1 h of incubation of the cytokine containing supernatant media with NPWT materials. All bar plots are represented as the mean of 3 independent experiments, with error indicating the S.E.M. Abbreviations are as follows: UL—Ultralow attachment plate surface, VC –Veraflo Cleanse, GF—Granufoam, CT—Cotton gauze, GL—Glass coverslip. * indicates *p* < 0.05 as tested using one-way ANOVA with multiple comparisons.

We next asked if the NPWT materials were adsorbing (and possibly absorbing) some of the cytokines produced by macrophages. To help answer this, we quantified TNF-α that was physically retained in the materials using a modified ELISA protocol. We found that while VC or GF did not retain appreciable quantities of TNF-α, CT was found to store a significant quantity of TNF-α (Figure 5a). This experiment suggested that neither of the NPWT materials were sequestering TNF-α, making it unlikely that is a major mechanism of macrophage inflammatory suppression.

We next tested the possibility that some of the inflammatory modulation observed for NPWT materials could stem from LPS/IFNγ sequestration. In such a scenario, cells could be inflamed to a lesser degree, explaining part of the reduced TNF-α that cells exhibit when cultured on these materials. To test this directly, we pre-incubated LPS/IFNγ-containing media for an hour on these materials, before transferring the media over to macrophages. TNF-α secreted by the cells were then measured using an ELISA (Figure 5b). Interestingly, all three materials studied, VC, GF, and CT, sequestered LPS/IFNγ to varying degrees. VC removed much of the stimulating biomolecules from the media, causing macrophages to produce negligible TNF-α, while GF and CT were capable of sequestering some of the inflammatory stimulants, such as LPS/IFNγ, from the media. This highlights the effectiveness of these materials in reducing the bioburden from wound exudates that might otherwise cause runaway inflammatory responses by the wound immune cells.

Subsequently, we assessed if the materials were sequestering cytokines, which could explain the reduced concentration in the media. Such a scenario represents an inability of the cytokines to act as soluble signaling molecules for potential supplemental stimulation. This experiment involved transferring the cytokines that the macrophages secrete under traditional culture conditions onto the dressing materials for an hour. ELISA was then performed to measure the TNF-α levels in the media (Figure 5c). Interestingly, while neither GF nor CT was capable of significant sequestration of TNF-α, VC was capable of sequestering TNF-α to undetectable levels in the media.

In our study, we found that certain analysis techniques cannot be easily performed on NPWT materials. Western blotting was difficult, given that all the materials absorbed a significant quantity of cell lysis buffer. Immunofluorescence staining of NPWT samples were not feasible for a few reasons. We found that both GF and VC exhibit significant autofluorescence across the spectrum (Figure S3). In addition, cryo-sectioning or paraffin-embedded sectioning of NPWT samples was not viable because the highly porous materials collapse when sectioned. Lastly, cotton adsorbed (and possibly absorbed) DAPI and secondary antibodies, leaving a significant background signal. Brightfield imaging of the cells was also not possible at higher magnifications (10× or higher), owing to the opacity of the materials. Given the difficulty in imaging the cells directly, we were unable to make direct comparisons of the morphology of cells on these materials. Despite these limitations, we underscore the importance of studying the effects of these materials on the cells in vitro to explain the molecular mechanisms of immunomodulation that happen clinically.

## 4. Conclusions

NPWT/vacuum-assisted closure of wounds has been utilized in various forms clinically for over two decades now. Its utility in chronic non-healing wounds and burns suggests it might play a role in suppressing inflammation at the wound site. Previous studies have indicated the presence of lower inflammatory cytokines with NPWT [16]. In addition, vacuum has been known to cause reduced inflammation and autophagy in macrophages [17]. Much of these studies have been performed in vivo. Given macrophage plasticity and mechano-responsiveness to their substratum, we asked if the NPWT materials themselves could play a role in suppressing inflammation in macrophages. We compared the capabilities of two NPWT materials, GF and VC, in modulating the macrophage inflammatory response, using CT as an absorbent wound dressing control, UL as a non-adhesive surface control, and GL as a highly cell-adhesive surface control.

We found that GF and VC NPWT materials modulate macrophages in different ways. SEM images of the macrophage morphology suggest that VC does not support macrophage adhesion or spreading as much as GF does. This can be explained by the increased hydrophilicity of VC. In addition, VC is capable of sequestering LPS/IFNγ, effectively, suppressing soluble cues that enable macrophage inflammatory polarization. VC may also sequester proinflammatory cytokines, such as TNF-α, if the cells happen to produce them. This leads to suppression of the possible supplemental inflammatory stimulation. Given that VC is used as an NPWT material with instillation, the ability of VC to sequester inflammatory cytokines, such as TNF-α, may play favorably to eliminate bioburden and alleviate inflammatory signals from the wound bed. GF, on the other hand, suppresses inflammation mainly by LPS/IFNγ sequestration. Compared to VC, it permits macrophage adhesion and spread to a greater degree. CT controls were also suppressive of the inflammatory genes *TNF* and *IL1B*, especially when cells were in direct contact with the material. Our study on macrophage inflammatory suppression in NPWT materials as culture substrates highlights the importance of in vitro studies that untangle the effects of vacuum-mediated hypoxia and substrate-mediated immunomodulation on cells.

## Data Availability

The data presented in this study are available on request from the corresponding author.

## Supplementary Materials

The following are available online at https://www.mdpi.com/article/10.3390/bioengineering9010002/s1, Figure S1: Summary of material properties used in the study, and the results of MTT assay used to assess the cytotoxicity of the materials, Figure S2: Counts of cells measured on the plate and media in the CyQUANT assay, Figure S3: Evaluation of material autofluorescence using epifluorescence microscopy.
