# Supplementary material for: Characterization of Macrophage and Cytokine Interactions with Biomaterials Used in Negative-Pressure Wound Therapy
Source: Bioengineering (Basel). 2021 Dec 22;9(1):2. doi: 10.3390/bioengineering9010002 (PMC8773312; doi:10.3390/bioengineering9010002)
Supplement: Supplementary file 1 [file bioengineering-09-00002-s001.zip › bioengineering-1507572-supplementary.pdf]

	Granufoam (GF)	Veraflo Cleanse (VC)	Cotton (CT)
Appearance and composition	Black, polyurethane ether, open cell reticulated foam	Gray, polyurethane ester, open cell reticulated foam	White, woven cotton gauze pads (majorly cellulose)
Porosity	400 - 600 μm Isometric pores	133 - 600 μm Anisometric pores	N/A
Hydro-phobicity	Relatively hydrophobic compared to VC	Relatively hydrophilic compared to GF	Highly absorbent
Application	NPWT without instillation	NPWT with instillation	Control; wound contact layer
Polymer chemical structure	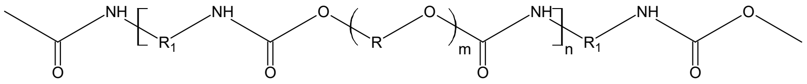 Polyurethane ether 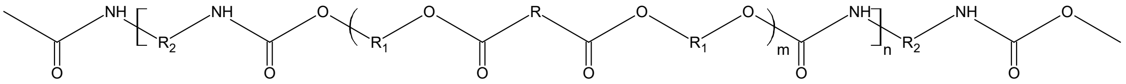 Polyurethane ester 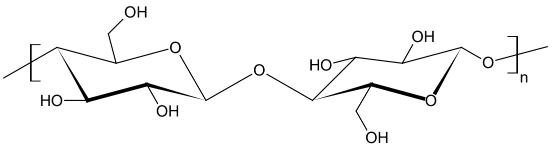 Cellulose		

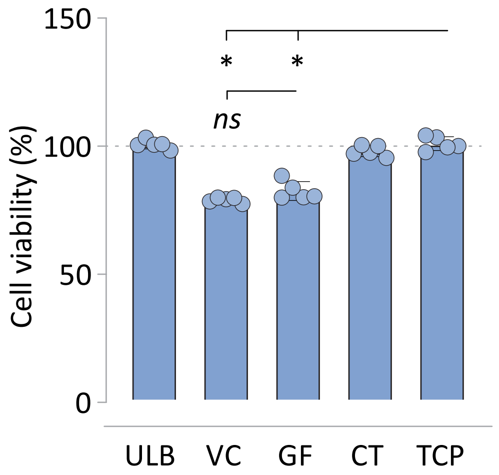

Figure S1. Summary of material properties used in the study (above), and the results of MTT assay used to assess the cytotoxicity of the materials (below, results of 5 replicate experiments). Abbreviations are as follows: UL – Ultralow attachment plate surface, VC – Veraflo Cleanse, GF – Granufoam, CT – Cotton gauze, TCP – Tissue culture plastic. * indicates $p < 0.05$ as tested using one-way ANOVA with multiple comparisons.

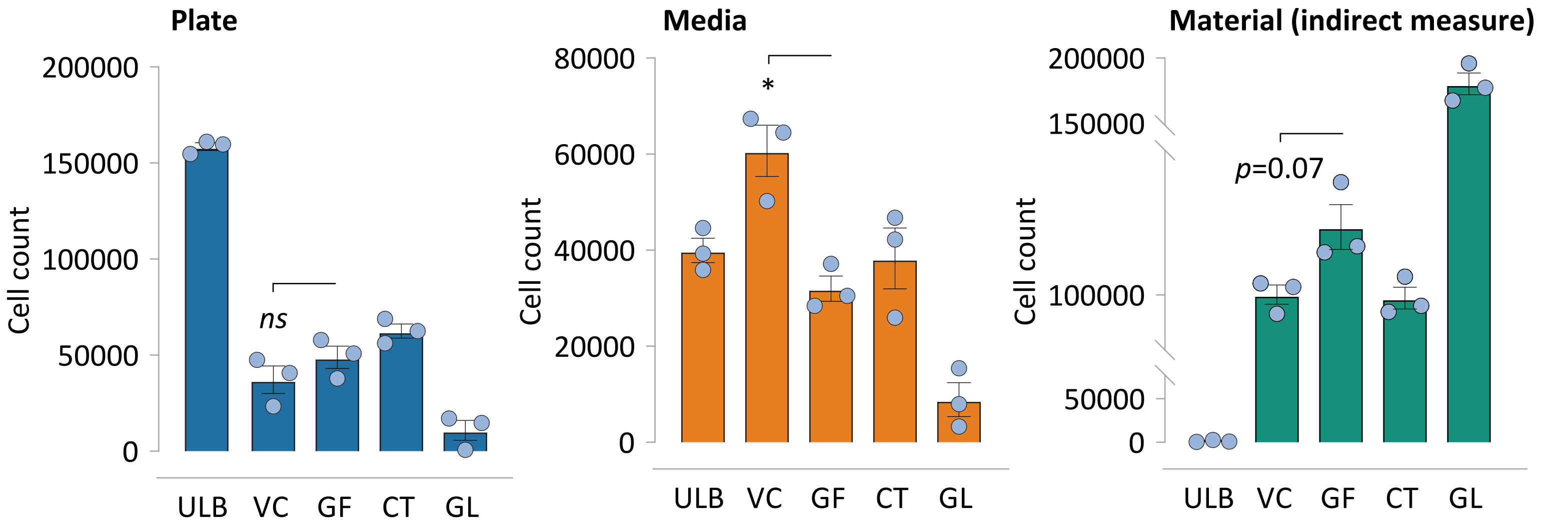

Figure S2. Counts of cells measured on the plate and media in the CyQUANT assay in figure 2. The media had a significantly higher number of cells when cultured on VC, in comparison with all other conditions, including GF. All bar plots are represented as mean of 3 independent experiments, with error indicating the S.E.M. Abbreviations are as follows: UL – Ultralow attachment plate surface, VC – Veraflo Cleanse, GF – Granufoam, CT – Cotton gauze, GL – Glass coverslip. * indicates $p < 0.05$ as tested using one-way ANOVA with multiple comparisons.

Supplemental Figure 3

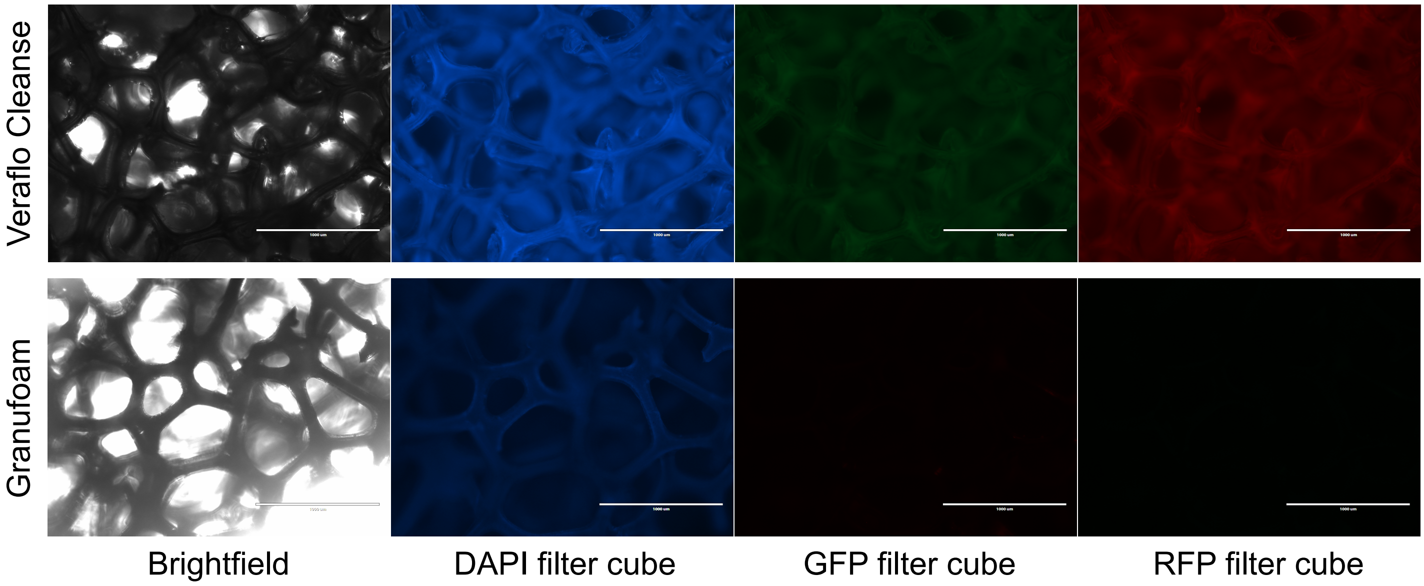

Figure S3. Evaluation of material autofluorescence using epifluorescence microscopy.
